# Structure and functional analyses of vaccinia virus J5 protein reveal distinct determinants for entry-fusion complex assembly and activation

**DOI:** 10.1128/jvi.00379-26

**Published:** 2026-07-06

**Authors:** Hsiao-Jung Chiu, Kathleen Joyce Carillo, Louise Tzung-Harn Hsieh, Yuan-Chao Lou, Chang Sheng-Huei Lin, Der-Lii Tzou, Wen Chang

**Affiliations:** 1 Molecular and Cell Biology, Taiwan International Graduate Program, Academia Sinica and Graduate Institute of Life Sciences, National Defense Medical University 71548, Taipei, Taiwan, ROC; 2 Institute of Molecular Biology, Academia Sinica 38017, Taipei, Taiwan, ROC; 3 Institute of Chemistry, Academia Sinica 38017, Taipei, Taiwan, ROC; 4 Biomedical Translation Research Center, Academia Sinica 38017, Taipei, Taiwan, ROC; Northwestern University Feinberg School of Medicine, Chicago, Illinois, USA

**Keywords:** virus entry, J5 protein, entry-fusion complex, vaccinia virus

## Abstract

Vaccinia virus enters host cells through a multi-component entry-fusion complex (EFC) that is structurally distinct from canonical viral fusion systems. Understanding how vaccinia virus EFC mediates membrane fusion is crucial for elucidating poxvirus entry and identifying potential antiviral targets. Here, we report the solution NMR structure of a truncated ectodomain of vaccinia J5 protein, residues 2–68. Using recombinant vaccinia viruses expressing J5 mutants, we analyzed substitutions in conserved and surface-exposed residues, as well as chimeric constructs between vaccinia J5 and its entomopoxvirus ortholog AMV232. Functional analyses revealed that the conserved P^38^YYCWY^43^ motif is dispensable for EFC assembly but required for membrane fusion activity, whereas the flexible region spanning residues 90–110 mediates interactions required for stable incorporation of J5 into the EFC.

**IMPORTANCE:**

Vaccinia virus enters host cells through membrane fusion mediated by a unique multi-component entry-fusion complex (EFC) that is distinct from classical viral fusion proteins. Although J5 has been identified as a central component of the pre-fusion EFC, the structural regions of J5 required for membrane fusion remain unclear. Here, we determined the solution NMR structure of the J5 ectodomain and identified two regions, the conserved P^38^YYCWY^43^ motif and residues 90–110, as key determinants of EFC function during vaccinia virus entry.

## INTRODUCTION

Vaccinia virus (VACV) is a prototypic member of the family *Poxviridae* and contains a linear, double-stranded DNA genome of approximately 194.7 kilobase pairs, encoding more than 200 open reading frames (1, 2). VACV has served as a model virus for studying poxvirus molecular biology and virus–host interactions. Historically, vaccinia virus was used as the live vaccine that led to the global eradication of smallpox caused by variola virus (3). More recently, it has also been employed as a vaccine platform to prevent infection with emerging orthopoxviruses, such as monkeypox virus (Mpox), during recent outbreaks (4).

VACV has a broad host range and completes its virus life cycle in the cytoplasm of host cells (5). Upon infection and subsequent core uncoating, viral factories form in the perinuclear regions of the cytoplasm and further assemble into mature virus (MV) containing a single membrane derived from the endoplasmic reticulum (6–9). A portion of these infectious particles is then transported to the Golgi cisternae, where additional membrane wrapping occurs (10, 11), and the wrapped virus subsequently egresses as a double-membrane extracellular virus (EV) (12–14).

Although MVs and EVs mediate distinct modes of transmission and enter host cells via different mechanisms (15–17), both forms of viral particles utilize an identical multiprotein (18), highly conserved entry-fusion complex (EFC) for membrane fusion between viral and host membranes. The EFC contains 11 component proteins: A16 (19), A21 (20), A28 (21), F9 (22), G3 (23), G9 (24), H2 (25), J5 (26), L1 (27), L5 (28), and O3 (29). Unlike the vast majority of viruses that utilize a single viral protein to engage cellular receptors and subsequently trigger membrane fusion through conformational changes (30), poxviruses instead employ multiple proteins. At least four viral proteins mediate attachment to glycosaminoglycans and laminin on the cell surface (31–34). In addition, each EFC component is pivotal for the viral infectivity, EFC assembly, and membrane fusion activity, although individual proteins may function at distinct stages of the fusion process (35).

Structures of individual EFC components have been determined, including A21 (36), A28 (37), F9 (38), H2 (39), L1 (40), the subcomplexes A16/G9 (41), and G3/L5 (42), and most recently the complete EFC (43). However, none of these structures or protein sequences exhibit detectable homology to well-characterized fusion proteins (44) of the classes I, II, or III (45, 46), suggesting that VACV employs a distinct membrane fusion mechanism.

Among the VACV EFC components, two structurally conserved paralogous groups have been identified: the A16/G9/J5 paralogs and F9/L1 paralogs (47, 48). These families share conserved cysteine positions and disulfide bonding patterns, which are widely distributed across the *Poxviridae* family (48), suggesting a conserved fusion mechanism during evolution. J5 is the shortest protein within the evolutionarily conserved A16/G9/J5 paralogs (26). Attempts to generate a J5 knockout virus were unsuccessful, indicating that J5 is essential for virus growth in cells (49). J5 associates with G9 and A16 via the conserved PXXCW and Delta motifs, thereby stabilizing the central core in the cryo-EM structure of the EFC (43). However, the specific residues of J5 that regulate membrane fusion remain poorly defined. In this study, we determined the solution nuclear magnetic resonance (NMR) structure of a truncated J5 ectodomain protein. Through mutagenesis and recombinant virus construction, we identified critical residues in J5 required for membrane fusion and EFC assembly, providing new insights into the functional architecture of the vaccinia virus entry-fusion complex.

## MATERIALS AND METHODS

### VACV J5 plasmid construction, protein expression, and purification

The full-length J5 (residues 1–133) was predicted to contain a long-disordered loop at its C-terminus preceding its single-pass transmembrane domain. Initial attempts to express the full-length J5 alone in soluble form were unsuccessful. The cDNA encoding the soluble fragment of the vaccinia virus J5 protein (residues 2–68; designated tJ5) was cloned into the pETDuet-1 vector with an N-terminal His₆-SUMO (Smt3) fusion tag under the control of the T7 promoter. The construct was transformed into *E. coli* BL21(DE3) cells for protein expression. For isotopic labeling, uniformly labeled ^15^N/^13^C protein was expressed in M9 minimal medium containing 1 g/L ^15^NH_4_Cl (Sigma-Aldrich) as the sole nitrogen source and 1 g/L uniformly labeled ^13^C_6_ glucose (Cambridge Isotope Laboratories) as the sole carbon source. The medium was supplemented with 2 mM MgSO_4_, 0.1 mM CaCl_2_, 1 mg/L thiamin, and 1 mg/L biotin. Cells were initially grown overnight in 30 mL of LB medium and then transferred to 1 L of M9 medium after centrifugation to remove residual LB medium. Protein expression was induced by adding 0.5 mM isopropyl-β-d-thiogalactoside (IPTG) when the culture reached an optical density at 600 nm (OD_600_) of 0.6. Cells were incubated for 18 h at 16°C and harvested by centrifugation at 3,000 × *g* for 30 min at 4°C.

Cell pellets were resuspended in lysis buffer containing 20 mM Tris (pH 8.0), 50 mM NaCl, 1 mM phenylmethylsulfonyl fluoride (PMSF), 1 mM 1,4-dithiothreitol (DTT), and lysozyme until homogeneous. Cell lysis was performed by sonication on ice. The lysate was cleared by centrifugation at 30,000 × *g* for 1 h at 4°C and filtered through a 0.45 μm filter. The clarified supernatant was incubated overnight at 4°C with 1 mL Ni^2+^-NTA affinity resin (Bio-Rad) packed into an empty PD10 column. The resin was washed three times with 10 column volumes of 20 mM Tris (pH 8.0) containing 500 mM NaCl and 20 mM imidazole. The protein was then eluted with 20 mM Tris (pH 8.0), 500 mM NaCl, 500 mM imidazole, and 1 mM DTT. To remove the His_6_-SUMO tag, Ulp1 protease (50) was added during overnight dialysis in 20 mM Tris (pH 8.0) and 50 mM NaCl at 4°C. Untagged tJ5 protein was subsequently separated using Ni-NTA resin (Bio-Rad). The protein sample was concentrated to 0.5 mM using an Amicon Ultra Centrifugal Filter with a 3-kDa molecular weight cutoff (Millipore). The concentrated protein was then exchanged into NMR buffer containing 10 mM phosphate (pH 6.5), 137 mM NaCl, and 2.7 mM KCl. Protein concentration was determined by absorbance at 280 nm (ε280 = 13,325 M^−1^ cm^−1^), and purity was confirmed by 15% SDS-polyacrylamide gel electrophoresis (SDS-PAGE).

### NMR spectroscopy and structural determination

All NMR spectra were recorded at 298 K and pH 6.5 using a Bruker AVANCE 600- or 800-MHz spectrometer equipped with a 5-mm triple-resonance TXI cryogenic probe with a shielded Z-gradient. Samples of 0.5 mM ^15^N/^13^C-labeled tJ5 in 1× PBS buffer (90% H_2_O and 10% D_2_O at pH 6.5) were loaded into 5-mm Shigemi NMR tubes for the experiments.

Sequential backbone resonance assignments were obtained through independent connectivity analysis of NHCACB, CBCA(CO)NH, HNCO, and HN(CA)CO experiments. NOE restraints were derived from 3D ^13^C- and ^15^N-edited NOESY-HSQC spectra with a mixing time of 150 ms. Backbone assignments and NOE resonances were analyzed using Topspin 4.3.0 (Bruker) and CARA 1.8.4 (51). NOE cross-peaks were categorized as very weak, weak, medium, or strong based on signal intensity. Hydrogen bond restraints were identified by monitoring slow HN exchange with solvent D_2_O. Dihedral angle restraints (ϕ and Ψ) were predicted from chemical shift data using the TALOS+ web server (52). For the final set of protein structure calculations, 100 tJ5 structures were generated using XPLOR-NIH 3.4 (53, 54) with a standard simulated annealing protocol, including high-temperature dynamics at 1,000 K followed by cooling to 100 K. The 20 lowest-energy structures were selected for water refinement using the AMPS-NMR web portal (55) and a standard restrained molecular dynamics protocol implemented within the AMBER99SB-ILDN force field (56). Refinement employed a generalized Born solvent model with an ionic strength of 137 mM NaCl and a 10 Å TIP3P water box. The final ensemble of 10 structures contained no distance or dihedral angle violations exceeding 0.5 Å or 5°, respectively. Structural quality was assessed using the Protein Structure Validation Suite (PSVS) (57). Protein structure figures were generated using the UCSF Chimera 1.18 (58). Statistics for the final structure are summarized in Table 1. The coordinates for the 10 conformers of tJ5 have been deposited in the Protein Data Bank under accession code 8WT5.

**TABLE 1:** NMR and refinement statistics for vaccinia virus fusion protein tJ5

Parameter	Value for tJ5 (2–68)
NMR distance and dihedral constraints	
Distance constraints	
Total NOE	836
Intraresidue	294
Interresidue	543
Sequence (|*i−j|* = 1)	294
Medium range (|*i−j|* ≤ 4)	296
Long range (|*i−j|* ≥ 5)	119
Dihedral angle restraints	78
*ϕ*	39
*φ*	39
Hydrogen bond restraints*^a^*	127
Structure statistics	
Distance violations per structure	
0.1–0.2 Å	6.1
0.2–0.5 Å	10.0
>0.5 Å	8.3
r.m.s. of distance violation per constraint	0.1 Å
Max. distance violation	1.55 Å
Dihedral angle violations per structure	
1–5°	6.3
>5°	5.6
r.m.s. of dihedral violation per constraint	1.0°
Max. dihedral angle violations	19.3°
Ramachandran plot summary*^b^*	
Most favored regions	98%
Additionally allowed regions	1%
Generally allowed regions	1%
Disallowed regions	0%
Averaged r.m.s.d. to the mean structure*^c^*	
Backbone	0.41 Å
Heavy atoms	0.45 Å

^a^ Two distance ranges (NH-O = 1.8–2.4 Å, N-O = 2.8–3.4 Å) were used for the hydrogen bond restraints between the amide and carbonyl group atoms.

^b^ Ramachandran plot analysis was performed over residues 2–68 with PROCHECK.

^c^ Average r.m.s.d. was calculated from an ensemble of 10 structures over secondary structure elements, including α-helices: residues 3–12, 39–42, 44–47, and 55–64.

### Cells, viruses, and reagents

BSC40, CV-1, and GFP- and RFP-expressing HeLa cells were cultured in Dulbecco’s modified Eagle’s medium (DMEM) supplemented with 10% fetal bovine serum (HyClone), penicillin (100 units/mL), and streptomycin (100 µg/mL) (Gibco) (59). The Western Reserve (WR) strain of vaccinia virus and recombinant viruses constructed in this study were propagated in BSC40 cells. Restriction enzymes were purchased from New England Biolabs. Fixative and staining solutions for electron microscopy analysis were obtained from Electron Microscopy Sciences.

### Bioinformatic analyses of poxviral J5 orthologs

Orthologs of the vaccinia J5 protein from representative members of the family *Poxviridae* were retrieved from GenBank with the following gene accession numbers: YP_232979.1 (vaccinia virus, VACV), NP_570485.1 (camelpox virus, CMLV), NP_619893.1 (cowpox virus, CPXV), NP_671599.1 (ectromelia virus, ECTV), NP_536516.1 (monkeypox virus, MPXV), YP_009282790.1 (skunkpox virus, SKPV), YP_717406.1 (taterapox virus, TATV), NP_042126.1 (variola virus, VARV), YP_009281844.1 (volepox virus, VPXV), NP_039099.1 (fowlpox virus, FWPV), YP_009177120.1 (turkeypox virus, TKPV), YP_001293261.1 (goatpox virus, GTPV), YP_004821430.1 (yokapox virus, YKPV), QGT49348.1 (crocodilepox virus, CRPV), YP_008658487.1 (squirrelpox virus, SQPV), NP_051781.1 (myxoma virus, MYXV), NP_044029.1 (molluscum contagiosum virus, MCPV), YP_009480606.1 (sea otterpox virus, SEPV), NP_957964.1 (bovine papular stomatitis virus, BPSV), YP_009112794.1 (red deer parapoxvirus, RDPV), YP_003457360.1 (pseudocowpox virus, PCPV), NP_957832.1 (orf virus, ORFV), YP_009268788.1 (pteropox virus, PTPV), NP_938325.1 (yabapox virus, YMTV), YP_009001515.1 (anomalacuprea entomopoxvirus, ACEV), YP_009408025.1 (eptesipox virus, EPTV), and NP_065014.1 (Amsacta moorei entomopoxvirus, AMV). Multiple sequence alignments were generated using MAFFT v7.453 with parameters --maxiterate 1000 --globalpair, and visualized with Jalview v1.8.3. Residue conservation was color-coded in blocks as follows: yellow (identical), blue (>0.5 conservation), and green (>0.2 conservation). Protein sequences of VACV J5 and AMV232 were further aligned using the ClustalW algorithm in MacVector (v16.0.10) with identical residues shaded in gray.

### Protein structure prediction with AlphaFold2

The protein structures of the swap mutations were predicted using AlphaFold2 (https://reurl.cc/96anZj). Sequence searches were performed using MMseqs2 (mmseqs2_uniref_env) in unpaired/paired mode. For each protein, the predicted model with the highest overall confidence (pLDDT >70) was selected for analysis. The pLDDT score for each model is shown in the corresponding figure.

### Structural alignment and residue interaction analysis

Protein structural similarity was analyzed in UCSF Chimera (version 1.18). Structural superimposition was performed using the Matchmaker tool, which first generates a sequence alignment followed by structural fitting of aligned residue pairs. Default alignment parameters were applied, including the Needleman-Wunsch algorithm, with the alignment score calculated as 70% residue similarity based on the BLOSUM62 substitution matrix and 30% secondary-structure similarity. Structural fitting was performed using Cα atoms of aligned residue pairs. Root mean square deviation (RMSD) values were calculated using the Match-Align function following structural superposition. Residue interaction analysis of the EFC (PDB 9UZO) was performed using the Residue Interaction Network Generator (RING; https://ring.biocomputingup.it/) and PyMOL to identify intermolecular contacts.

### Construction of J5 mutant plasmids and recombinant vaccinia viruses

Recombinant J5 vaccinia viruses were constructed by homologous recombination between the viral genome and pBlueScript-based transfer plasmids (pBlueScript-J6R-J5L-gpt-J4R-J3) carrying WT or mutant *J5L* ORFs, followed by gpt selection (60). Briefly, a 1 kb flanking fragment containing a partial J6R sequence and the J5L promoter region was amplified from WR genomic DNA using the primers 5′-TAGGGCGAATTGGGTACCAACGGTGATAGATGTACT-3′ and 5′-GAATTCGATATCAAGCTTGTATACCTTCGGTTCTTT-3′ (the *Kpn*I and *Hind*III sites are underlined) and cloned into pBlueScript KS(+). Then, a mutation was introduced to disrupt a *Bst*Z17I site using the primers 5′-AATTCATTGGTTTCTTTGGG**A**ATACTATCTATCCAAAAACT and 5′-AGTTTTTGGATAGATAGTAT**T**CCCAAAGAAACCAATGAATT-3′ (the mutated *Bst*Z17I site is underlined with the mutant nucleotide bolded), generating pBlueScript-J6R.

The Eco*Gpt* cassette and 1 kb downstream flanking fragment containing the *J4R* ORF and partial *J3R* sequences were generated by PCR using the primers 5′-CAAGCTTGATATCGAATTCAATTGGATCACTAATTCCAAACC-3′ and 5′-TATTTCAGTCGCTGATTAATCTAGCGACCGGAGATTGGCGG-3′, 5′-CCGCCAATCTCCGGTCGCTAGATTAATCAGCGACTGAAATA-3′ and 5′-TGGAGCTCCACCGCGGTGGCGGCCGCCCGTTTCCAGATCAATGGAT-3′, respectively, and assembled with linearized pBlueScript-J6 using NEBuilder to generate pBlueScript-J6R-gpt-J4R-J3.

WT and mutant *J5L* ORFs were synthesized (GenScript Inc.) with *Bst*Z17I and *Mfe*I sites at their 5′ and 3′ ends, respectively, and subcloned into pBlueScript-J6R-gpt-J4R-J3 by restriction enzyme digestion and ligation, yielding a set of J5 transfer plasmids, designated pBlueScript-J6R-J5L (WT or mutant)-gpt-J4R-J3 (Table S1 contains mutant J5 protein sequences translated from each synthetic ORF). In some cases, *J5L* mutants were generated from the WT *J5L* transfer plasmid using the QuickChange Lightning site-directed mutagenesis kit (Agilent Technologies) with the primers listed in Table S2.

Finally, to generate recombinant VACV, CV-1 cells were infected with wild-type VACV at a multiplicity of infection (MOI) of 1 PFU per cell and subsequently transfected with individual J5 transfer plasmids expressing either wild-type or mutant J5 protein as described above. The infected cells were harvested at 24 h post-infection (hpi), and recombinant viruses were isolated by at least three rounds of plaque purification in 1% agar under *gpt* selection medium containing 25 µg/mL mycophenolic acid, 250 µg/mL xanthine, and 15 µg/mL hypoxanthine, as described previously (60).

### Viral genome sequencing analysis

Vaccinia viral genomic DNA isolation and sequencing analysis were performed with minor modifications from previously described methods (61, 62). Briefly, BSC40 cells were infected with WT or J5 mutant recombinant viruses at an MOI of 5 PFU per cell and harvested at 18–20 hpi. Viral genomic DNA was isolated and quantified using Qubit dsDNA assays.

For whole-genome sequencing, viral DNA libraries were prepared using either Illumina or Oxford Nanopore Technologies (ONT) sequencing platforms. Illumina libraries were prepared using the KAPA HyperPrep Kit and sequenced on an Illumina NovaSeq X Plus system with 150-bp paired-end reads. ONT libraries were prepared using the Native Barcoding Kit 24 V14 and sequenced on a PromethION platform. Sequencing was performed by Genomics BioSci & Tech Co., Ltd.

Sequencing reads were analyzed by alignment to the VACV WR reference genome (NC_006998). Host-derived reads were removed by alignment to the *Chlorocebus aethiops* reference genome, where applicable. Viral genome assemblies or consensus sequences were generated using standard read-mapping and assembly tools, including BWA-MEM (63), Samtools (64), SPAdes (65), minimap2, medaka, and MAFFT (66). The resulting viral genome sequences were examined to verify the intended J5L-gpt insertion and to identify possible second-site mutations. This analysis confirmed the correct incorporation of the WT or mutant J5L-gpt cassette in the recombinant viruses. No secondary mutations were detected within any ORF encoding the EFC components.

### Determination of J5 mutant virus infectivity

To measure virus infectivity, BSC40 cells were seeded in six-well plates and infected with J5 WT or J5 mutant recombinant viruses at an MOI of 5 PFU per cell. After 1 h of adsorption at 37°C, cells were washed with PBS and cultured in fresh growth medium for 24 h before harvesting. The viral titers of each sample were determined via plaque assays on BSC40 cell monolayers. Briefly, each sample lysate was serially diluted in PBS-AM buffer (PBS/0.05% BSA/1 mM MgCl₂) and used to infect BSC40 cells in six-well plates at 37°C for 1 h. After removing the virus inoculum, cells in each well were overlaid with 3 mL of DMEM supplemented with 10% FBS, 1% penicillin-streptomycin, and 1% agar, incubated at 37°C for 3 days, fixed, and stained with crystal violet. Plaques were visualized and counted to calculate the viral titers, which were expressed as plaque-forming units per milliliter (PFU/mL). All experiments were performed independently three times.

### MV-triggered cell–cell fusion at acid pH (fusion-from-without)

To evaluate the fusogenic activity of the J5 mutant viruses, vaccinia MV-mediated cell–cell fusion assays were performed as previously described (37, 39, 59). In brief, HeLa cells expressing either GFP or RFP were mixed at a 1:1 ratio and seeded at 3 × 10^4^ cells/well in a 96-well plate. Because J5 mutations do not affect virion morphogenesis (26) (Fig. S3), all the mutant viruses were expected to produce equivalent quantities of MV particles in infected cells. Consequently, normalization based on physical particle counts was achieved by standardizing the inoculum volume across all groups. Co-cultured HeLa cells were incubated with a volume of wild-type (WT) virus stock sufficient to achieve an MOI of 100 PFU/cell, and the same infection volume was used for all J5 mutant virus groups. The virus mixtures were incubated with cells at 37°C for 1 h. Cells were subsequently washed with PBS and treated with either acidic (pH 5.0) or neutral (pH 7.0) PBS at 37°C for 3 min to trigger membrane fusion and then replaced with fresh growth medium containing 40 μg/mL cordycepin. Cell fusion was monitored every 30 min using ImageXpress Confocal HT.ai High Content Imaging system (Molecular Devices) until 3 hpi. Fusion efficiency was quantified using Fiji software as follows: (surface area of GFP^+^RFP^+^ double-fluorescent cells/surface area of single-fluorescent cells) × 100% (37, 39, 59). All experiments were performed independently three times.

### Immunoblot analysis

Protein samples were denatured in SDS-containing sample buffer at 95°C for 10 min and analyzed by immunoblotting as previously described (37, 39, 59). In brief, samples were separated by 12% SDS-PAGE and transferred onto 0.45 µm nitrocellulose membranes (Bio-Rad). Viral proteins were detected using rabbit polyclonal antibodies generated previously: Antisera against A16, A21, A28, H2, G9, J5, L1, and D8 were produced by immunizing New Zealand White (NZW) rabbits with bacterially expressed soluble recombinant proteins (33, 37, 67), while anti-G3, anti-F9, and anti-L5 antibodies were raised against specific synthetic peptides (23, 67, 68). For immunoblotting, each primary antibody was diluted 1:1,000 (except 1:5,000 for D8) in 5% skim milk and incubated with the membranes at 4°C for 16–18 h. Following three washes with PBS-T (0.1% Tween-20), the membranes were incubated with HRP-conjugated secondary antibodies (goat anti-rabbit IgG [H + L]) (Thermo Fisher Scientific; 1:5,000) or EasyBlot anti-rabbit IgG (GeneTex; 1:5,000) for 1–2 h at room temperature, washed three times with PBS-T, and developed using an enhanced chemiluminescence substrate (PerkinElmer) and subsequently imaged using a UVP BioSpectrum 815 Imaging System (UVP, Upland, CA, USA).

### Coimmunoprecipitation

Confluent BSC40 cells were infected with recombinant J5 WT or mutant viruses at an MOI of 2 PFU per cell and incubated at 37°C for 24 h. Cells were washed with PBS, harvested, and lysed in ice-cold buffer containing 20 mM Tris (pH 8.0), 200 mM NaCl, 1 mM EDTA, and 1% NP-40 supplemented with protease inhibitors (1 mM phenylmethylsulfonyl fluoride, 2 µg/mL aprotinin, 1 µg/mL leupeptin, and 0.7 µg/mL pepstatin). After centrifugation to remove insoluble debris, 250 µg of the clarified lysate was incubated with anti-J5, anti-G9, or anti-A28 antisera at a 1:50 dilution in the presence of protein A-conjugated beads (GE Healthcare). The mixtures were rotated at 4°C for 16 h, centrifuged, washed five times with lysis buffer, and the bound proteins were subsequently separated on 12% SDS-PAGE and analyzed via immunoblotting as described above (37).

### Transmission electron microscopy analysis

The transmission electron microscopy (TEM) analyses were performed as previously described (62). In brief, confluent BSC40 cells were seeded onto poly-L-lysine-coated plastic slides and individually infected with J5 WT or mutant viruses at an MOI of 2 PFU per cell for 24 h. Cells were fixed in 2.5% glutaraldehyde, postfixed with 1% osmium tetroxide, and contrasted with 1% uranyl acetate. After serial ethanol dehydration, samples were embedded in Epon resin and sectioned for TEM. Samples were imaged on a Talos L120C transmission electron microscope equipped with a CETA 16 4k × 4k CMOS camera (Thermo Scientific).

### Statistical analysis

Statistical analysis was performed using GraphPad Prism v10 (GraphPad Software, San Diego, CA). Data are presented as mean ± standard deviation (SD). Statistical significance was assessed using a two-tailed Student’s *t* test. For comparisons with WT controls, the *P* value was adjusted using the “p.adjust” function with the false discovery rate (FDR) method in R v4.5.1. Adjusted *P* values of < 0.05 were considered statistically significant. Significance levels are indicated as follows: *, *P* < 0.05, **, *P* < 0.01; ***, *P* < 0.001,and ****, *P* < 0.0001.

## RESULTS

### NMR structure of truncated vaccinia virus fusion component J5 (tJ5)

**Fig 1:**
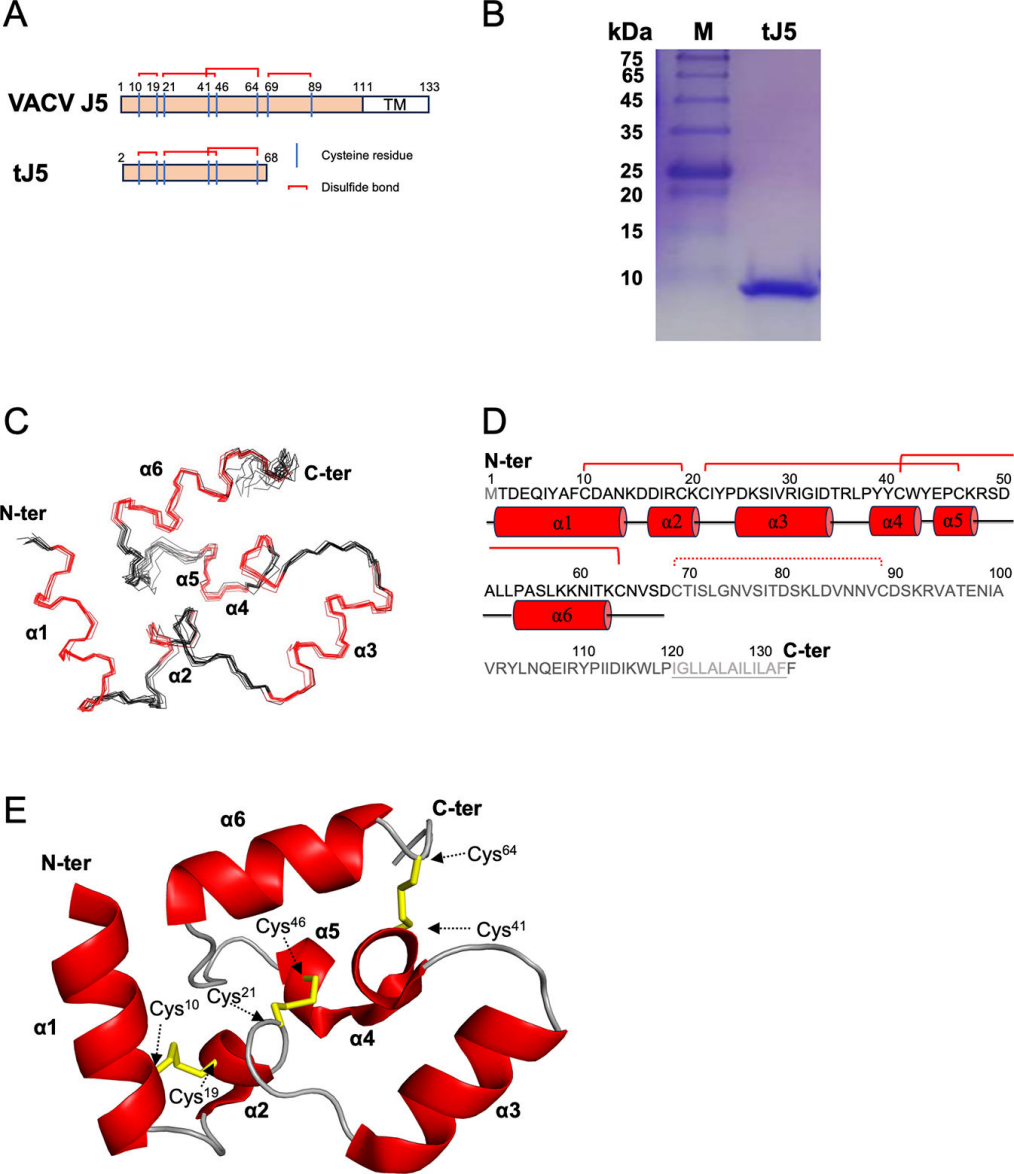
NMR structure determination of the vaccinia virus J5 ectodomain (tJ5, residues 2–68). (**A**) Schematic representation of the full-length J5 protein (residues 1–133) showing the transmembrane domain (TM; residues 111–133) and the truncated soluble construct (tJ5; residues 2–68) used for structural determination. The specific positions of conserved cysteine residues (blue bar) and the disulfide bonds (connecting red lines) are indicated. (**B**) SDS-PAGE analysis of purified recombinant tJ5 protein stained with Coomassie blue. M, protein markers (labeled in kDa). (**C**) An ensemble of the 10 lowest-energy NMR structures of tJ5 is represented as a bundle model. The ribbon is colored with α-helices in red and unstructured regions in black. (**D**) Secondary-structure organization of tJ5, consisting of six α-helices. Helices are represented as red cylinders; random coil regions are shown as black lines. The intramolecular disulfide bonds are shown as connecting lines between the paired cysteine residues. Residues present in the tJ5 construct are shown in black text. J5 residues absent from the construct are shown in gray, and transmembrane residues are underlined. (**E**) The lowest-energy NMR structure of tJ5 in ribbon format. Ribbon representation of the average NMR structure of tJ5, with α-helices shown in red and three pairs of disulfide bonds, C10-C19, C21-C46, and C41-C64, highlighted in yellow sticks.

We generated a bacterial expression plasmid encoding the ectodomain of vaccinia J5 protein (residues 2–68), designated tJ5, fused to a hexa-histidine tag at the N-terminus (Fig. 1A). The tJ5 protein was purified using Ni²^+^ affinity chromatography (Fig. 1B). Using ^15^N and ^13^C isotope labeling, we determined the backbone resonance assignments of tJ5 by three-dimensional heteronuclear NMR spectroscopy, as shown in the ^1^H-^15^N HSQC spectrum (Fig. S1). The 2D HSQC spectrum of isotopically labeled tJ5 displayed well-resolved resonances for most non-proline residues, suggesting that the protein is well-folded in solution. Secondary structure was predicted based on ^13^C_α_ chemical shift propensity analysis, which revealed six α-helices connected by loop regions within the N-terminal ectodomain (residues 2–68).

To determine the three-dimensional structure of tJ5, we performed multi-dimensional NMR experiments to obtain information on NOE-derived distance restraints and dihedral-angle restraints for protein structural calculations. During structural refinement, we applied the AMBER force field for energy minimization, incorporating explicit water molecules and ions to approximate the solution environment. These analyses yielded a high-resolution molecular structure with an average RMSD of 0.5 Å, as summarized in Table 1. The solution NMR structure of tJ5 consists of six α-helices, α1 (residues T2–N13), α2 (D16–K20), α3 (D25–D34), α4 (Y39–W42), α5 (E44–K47), and α6 (P54–K63) (Fig. 1C through E). Helices α1, α3, and α6 form a coplanar triangular ring structure, whereas the short helices α2, α4, and α5 project outward to create a central concave surface (Fig. 1E). This central region is stabilized by the C21-C46 disulfide bond and is further connected to the outer helices through two additional disulfide bonds: C10-C19 between α1 and α2, and C41-C64 between α4 and α6, resulting in a compact helical domain. Thus, the tJ5 construct captures the N-terminal folded ectodomain of J5, including three conserved disulfide bonds, but does not include the C-terminal portion of full-length J5. This structural arrangement suggests that these helices may provide a stable interaction surface for other EFC components and contribute to vaccinia virus infectivity.

**Fig 2:**
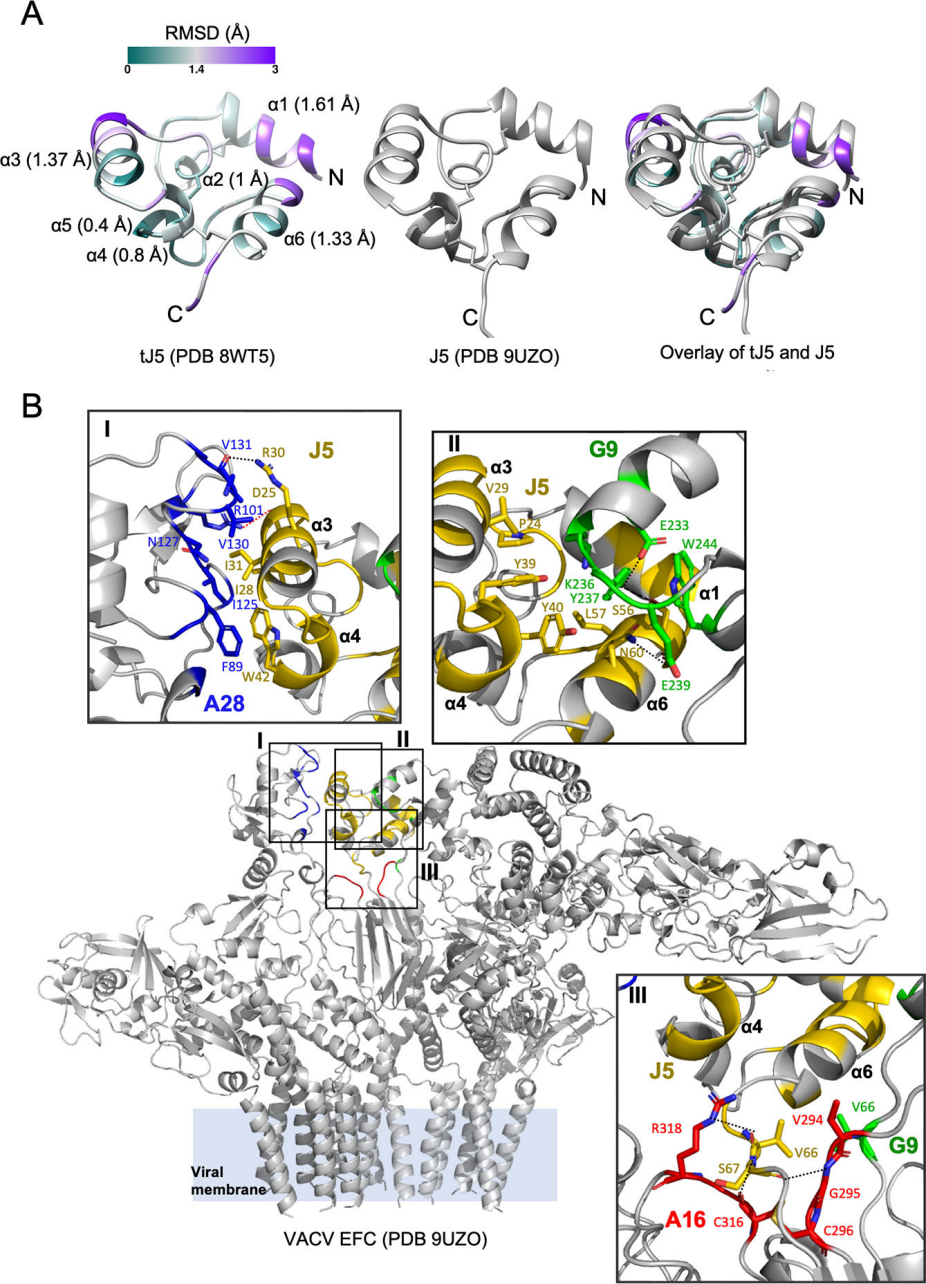
Structural alignment reveals conformational flexibility of α1, α3, α4, and α6 associated with J5 incorporation into the EFC. (**A**) Structural superimposition of tJ5 (PDB 8WT5, left) and J5 (PDB 9UZO, middle) with a comprehensive overlay model (right) generated in UCSF Chimera. RMSD values ranging from 0 to 3 Å, representing increasing structural deviation, are shown as a color gradient from teal to gray to purple and mapped onto the three-dimensional cartoon ribbons. The specific root-mean-square deviations calculated for discrete structural segments are labeled next to their corresponding domains: α1 (1.61 Å), α2 (1 Å), α3 (1.37 Å), α4 (0.8 Å), α5 (0.4 Å), and α6 (1.33 Å). (**B**) Cartoon representation of VACV EFC cryo-EM structure (PDB 9UZO) anchored to the viral membrane bilayer. Residues showing deviations greater than 1.5 Å between the solution structure of tJ5 and the complexed state of cryo-EM J5 are highlighted in yellow. Proteins located within 5 Å of J5 are colored as follows: A28 (blue), G9 (green), and A16 (red). Insets I, II, and III provide close-up magnifications of the discrete interactive interfaces: Inset I shows the interaction network between α3 and α4 of J5 (yellow) and adjacent loops of A28 (blue). Inset II demonstrates the hydrophobic contacts and hydrogen bonds between α1 and α6 of J5 (yellow) and the conserved PXXCW motif of G9 (green). Inset III shows the hydrogen bond interactions of the C-terminal α6 of J5 (yellow) with a loop located downstream of the Delta motif of A16. Residues involved in the contact interface are shown as sticks, with hydrogen bonds and salt bridges indicated by dashed lines and colored in black and red, respectively.

We next compared the solution NMR structure of tJ5 with the cryo-EM structure of J5 within the vaccinia virus EFC (43). Despite the overall conservation of spatial arrangement and disulfide bond positions, structural alignment yielded an overall RMSD of 1.176 Å across 60 aligned residues (T2–T62), with the largest deviation observed in helices α1 (1.61 Å), α3 (1.37 Å), and α6 (1.33 Å) (Fig. 2A). This comparison indicates that the N-terminal ectodomain observed by NMR adopts a similar overall fold within the full-length EFC structure, while the functional interpretation of regions outside residues 2–68 relies on the full-length EFC structure and virological analyses. Consistent with this moderate structural flexibility, J5 engages helices α3 and α4 to interact with the A28 protein, whereas helices α1 and α6 contact G9 and A16 within the assembled EFC (43). Specifically, helices α3 and α4 form hydrophobic interactions and hydrogen bonds with adjacent loops of A28 (Fig. 2B, inset I), whereas the α1 and α6 establish hydrophobic contacts and hydrogen bonds with the conserved PXXCW motif of G9 (Fig. 2B, inset II). In addition, the C-terminal region of α6 forms hydrogen bonds with a loop located downstream of the Delta motif of A16 (Fig. 2B, inset III). Together, these α-helical regions form a hydrophobic core while retaining structural flexibility, enabling selective protein–protein interactions with A28, G9, and A16 within the vaccinia EFC.

### Functional mapping of VACV J5 protein in virus infectivity using recombinant viruses

**Fig 3:**
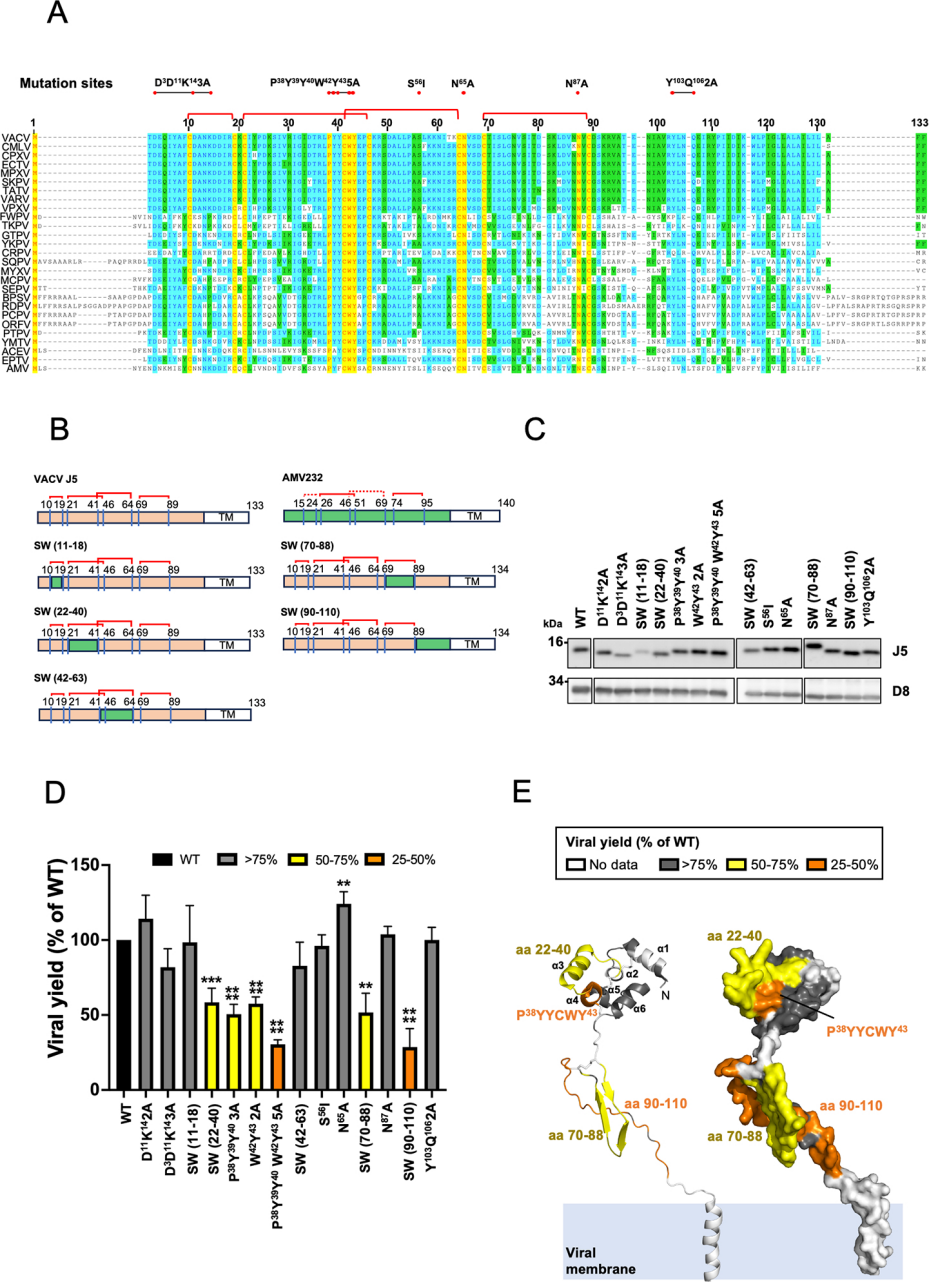
Functional mapping of the vaccinia virus J5 protein. (**A**) Multiple sequence alignment of J5 orthologs from 27 representative members of the family *Poxviridae*. Identical residues are shown in yellow (100% identity), moderately conserved residues in blue (>50%), and weakly conserved residues in green (>20%). The positions of the targeted mutation sites are indicated above the alignment. Predicted disulfide bonds are shown as red connecting lines between pairs of cysteine residues. (**B**) Schematic representation of full-length VACV J5 (residues 1–133, salmon red), entomopoxvirus ortholog AMV232 (residues 1–140, green), and five J5 swap mutants, SW (11–18), SW (22–40), SW (42–63), SW (70–88), and SW (90–110), with chimeric coloring indicating the sequence origin. (**C**) Immunoblot analysis of J5 and the control protein D8 in infected cell lysates across the complete panel of mutant viruses. (**D**) Virus yields of WT and mutant J5 recombinant viruses in BSC40 cells. Infectivity of each mutant was normalized to that of WT J5 and color-coded according to virus infectivity: gray bars (>75% yield), yellow bars (50%–75% yield), and orange bars (25%–50% yield). Data represent means ± standard deviations from three independent experiments; statistical significance levels relative to WT are indicated by asterisks (**, *P* < 0.01; ***, *P* < 0.001; and ****, *P* < 0.0001). (**E**) Mapping of virus yield loss onto the full-length J5 carton (left panel) and surface (right) models situated on the viral membrane. The locations of key functional regions, including residues 22–40 (α3), 70–88 (the Delta motif), 90–110, and the P^38^YYCWY^43^ motif, are labeled and color-coded accordingly.

To determine which residues of J5 are important for vaccinia virus infection, multiple sequence alignment of J5 orthologs from 27 members of the *Poxviridae* family was performed (Fig. 3A). A series of site-directed mutations was generated based on either surface-exposed charged residues (D3, D11, and K14) or conserved residues (P^38^YYCWY^43^, S56, N65, N87, and Y^103^Q^106^). In addition, all vaccinia J5 orthologs contain eight conserved cysteine residues predicted to form four disulfide bonds (Fig. 3A). The J5 ortholog in Amsacta moorei entomopoxvirus (AMV), AMV232, was selected as the swap target (Fig. 3B). Despite sharing low sequence identity with VACV J5 in our MSA analysis, AMV232 highly preserves the essential cysteine residues and disulfide-bond framework. Swapping sequences between these conserved cysteines allowed us to maximize the substitution of primary protein sequences to identify functionally critical motifs, while minimizing the risk of disrupting the overall structural integrity and stability of the chimeric J5 proteins. We therefore generated a set of five swap (SW) mutants, SW (11–18), SW (22–40), SW (42–63), and SW (70–88) each replacing the sequences between cysteine residues, and SW (90–110), which replaces the disordered loop preceding the transmembrane region (Fig. 3B; Fig. S2A). AlphaFold predicted that each of these SW mutant proteins retained an ectodomain structure (residues 2–68) similar to that of vaccinia WT J5 protein, with RMSD values of 0.886, 1.124, 1.122, 0.933, and 0.957 Å for SW (11–18), SW (22–40), SW (42–63), SW (70–88), and SW (90–110), respectively (Fig. S2B). These results suggest that the chimeric SW constructs likely maintain stable tertiary structures, allowing functional effects to be assessed without global structural disruption. While the helical ectodomains of all swap variants exhibited high structural reliability with RMSD values close to 1 Å, the structural models for SW (70–88) and SW (90–110) mutant proteins displayed relatively low prediction confidence (pLDDT <60) specifically within their flexible loop regions, indicating that these discrete loop segments may represent less reliable structural predictions (Fig. S2B).

Although deletion of *J5L* resulted in a lethal phenotype in vaccinia virus (26, 49), we generated recombinant viruses expressing WT or mutant J5 proteins by homologous recombination as described in Materials and Methods. In brief, the CV-1 cells were infected with WT vaccinia virus and transfected with individual plasmids encoding J5 mutant ORFs together with the *gpt* selection cassette, flanked by the 5′ and 3′ sequences of the *J5L* locus. A comprehensive list describing all J5 mutant construction strategies and the resulting amino acid sequences is detailed in Table S1. Recombinant viruses were isolated after three rounds of plaque purification in 1% agar under *gpt* selection, yielding 15 recombinant viruses, including a *gpt*^+^ virus expressing WT J5 protein. To exclude the possibility of second-site mutations in the recombinant viruses, viral genomic DNA was isolated from each recombinant virus and subjected to whole-genome sequencing (WGS) analysis; notably, no secondary mutations were detected within the J5L-gpt insertion locus or any other EFC ORFs.

We next evaluated the effect of these mutations on viral infectivity. BSC40 cells were infected at an MOI of 5 PFU/cell, and cell lysates harvested at 24 h post-infection were analyzed by plaque assay and immunoblotting. Expression levels of J5 and the control protein D8 were comparable among most mutants and the WT virus, except for the swap mutant SW (11–18), which exhibited reduced J5 protein abundance (Fig. 3C). This diminished signal may reflect either a genuinely lower expression level of the chimeric protein or impaired Ab reactivity toward the chimeric epitopes. Viral infectivity of WT J5 was normalized to 100% (Fig. 3D). Most J5 mutants retained >75% infectivity (gray bars), including SW (11–18). Four mutants, SW (22–40), P^38^Y^39^Y^40^3A, W^42^Y^43^2A, and SW (70-88), displayed moderate reductions in infectivity (50%–75%, yellow bars). Most importantly, two mutants, P^38^Y^39^Y^40^W^42^Y^43^5A and SW (90-110), showed the most profound loss of virus infectivity (25%–50%, orange bars).

We then map these functional regions onto the J5 structure to identify important structural features for J5 function. As the NMR J5 structure contains only the ectodomain, we mapped the functional regions to the full-length J5 structure of the EFC complex (Fig. 3E). These moderately impaired mutants SW (22–40), P^38^Y^39^Y^40^3A, and W^42^Y^43^2A map to helices α3 and α4 of J5 (Fig. 3E), whereas SW (70–88) comprises the Delta motif, a conserved disulfide-bonded closed loop within the A16/G9/J5 paralogs, that was shown to be critical for VACV infectivity and A16/G9 oligomerization (Fig. 3E) (59). Furthermore, the P^38^Y^39^Y^40^W^42^Y^43^5A mutant targets the conserved P^38^YYCWY^43^ motif shared among A16, G9, and J5 (P^262^RVCWL^267^ in A16; P^240^RECWD^245^ in G9; P^38^YYCWY^43^ in J5) (59), whereas the SW (90–110) mutant corresponds to a disordered loop located immediately preceding the transmembrane domain. Together, these infectivity assays indicate that mutations in helices α1, α2, α5, and α6 have relatively minor effects on viral infectivity, whereas mutations in helix α3 and the Delta motif result in moderate reductions in infectivity. In contrast, the P^38^YYCWY^43^ motif and the extended disordered loop spanning residues 90–110 are critical for maintaining EFC function and viral entry efficiency (Fig. 3E).

### Defective J5 mutants with altered P^38^YYCWY^43^ motif and disordered C-terminal loop (90–110) display reduced fusogenic activity

BSC40 cells infected with WT or mutant J5 viruses at 2 PFU/cell were fixed at 24 hpi and subjected to negative staining for TEM imaging analyses (Fig. S3). No obvious accumulation of immature particles or viral assembly intermediates was observed in any of the mutants, confirming that the viral morphogenesis pathway remains fully intact. Abundant intracellular mature virions with normal morphology were observed in cells infected with either WT or mutant viruses, consistent with previous findings that repression of EFC components does not affect virion morphogenesis or MV formation (18–29, 37).

**Fig 4:**
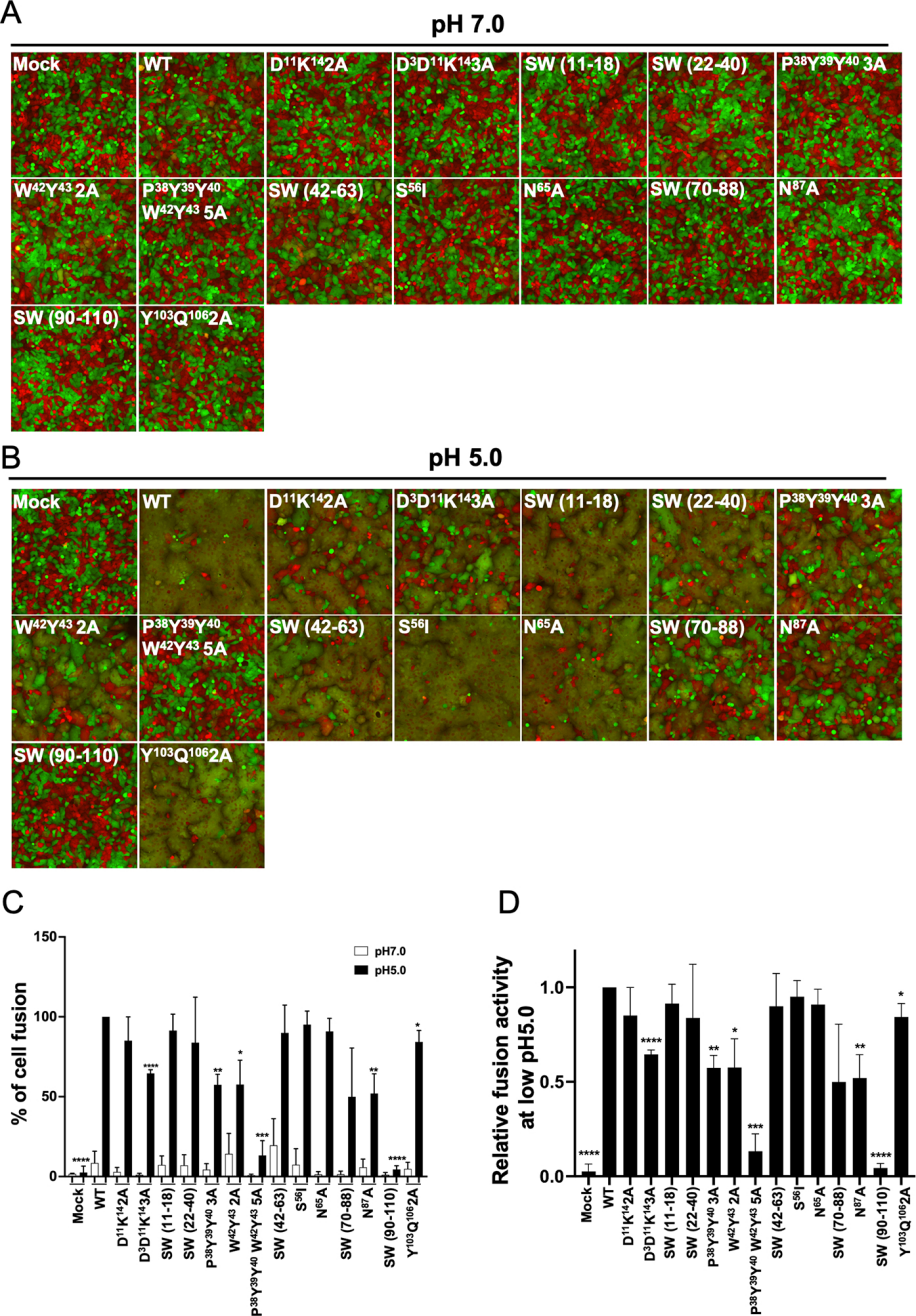
Cell–cell fusion assays of J5 mutants reveal impaired fusion activity at low pH. (**A**) Fluorescence imaging of HeLa cells expressing GFP or RFP was mixed at a 1:1 ratio and incubated with lysates containing WT or mutant J5 recombinant viruses under neutral pH conditions (pH 7.0). Fluorescence images were photographed at 3 hpi. (**B**) Parallel fusion assays were performed as described in panel A, except that cells were incubated in acidic buffer (pH 5.0) for 3 min to mimic endosomal acidification as described in Materials and Methods. (**C**) Quantification of MV-triggered cell–cell fusion under neutral or acidic conditions. Images from three independent experiments were analyzed using Fiji software. Fusion was calculated as the percentage of the surface area of double-fluorescent cells relative to that of single-fluorescent cells. White and black bars represent fusion rates at pH 7.0 and pH 5.0, respectively. (**D**) Relative fusion activity of each mutant normalized to WT at low pH. Data represent means ± standard deviations from three independent experiments. Statistical comparison of fusion was performed between J5 WT and each mutant using a two-tailed Student’s *t*-test. *P < 0.05; **P < 0.01; ***P < 0.001; and ****P < 0.0001.

We then investigated whether these J5 mutants had lost the ability to trigger membrane fusion, using a previously established MV-mediated cell-cell fusion assay (37, 39). Notably, the literature has shown that J5-depleted viruses produced a total MV particle yield comparable to that of the WT virus, despite exhibiting diminished infectivity (26). Therefore, as described in Materials and Methods, to ensure a stringent and biologically relevant comparison in our cell–cell fusion assays, the input virus quantities across all J5 mutants were strictly normalized to equal virus inoculum volumes rather than to PFU, thereby standardizing the physical particle input across all viruses. GFP- and RFP-expressing HeLa cells were mixed at a 1:1 ratio and infected at 37°C for 1 h with a volume of WT virus stock sufficient to achieve an MOI of 100 PFU/cell; an identical infection volume was used for all J5 mutant viruses. Following infection, cells were treated with either neutral (pH 7.0) or acidic (pH 5.0) buffer, washed, and subsequently maintained in growth medium. Cell fusion was monitored every 30 min for up to 3 hpi. Under neutral pH conditions, little or no fusion was observed for the WT or any J5 mutant viruses, as expected (Fig. 4A). In contrast, J5 WT induced robust syncytium formation following acid treatment, whereas the P^38^Y^39^Y^40^W^42^Y^43^5A mutant and the SW (90–110) mutant exhibited significantly reduced fusion when compared with the WT virus (Fig. 4B). The percentage of MV-induced cell fusion at each pH condition was individually determined (Fig. 4C) as described in Materials and Methods. We then calculated the relative fusion activity of each J5 mutant virus at pH 5.0 after normalizing the percentage of fusion of the WT virus to 100% activity (Fig. 4D) (62). Together, these data demonstrate that the conserved P^38^YYCWY^43^ motif and the disordered region spanning residues 90-110 are critical determinants required for J5-mediated membrane fusion during vaccinia virus entry.

### The P^38^YYCWY^43^ motif is dispensable for assembly but essential for function, whereas residues 90–110 are required for incorporation of J5 into the EFC

**Fig 5:**
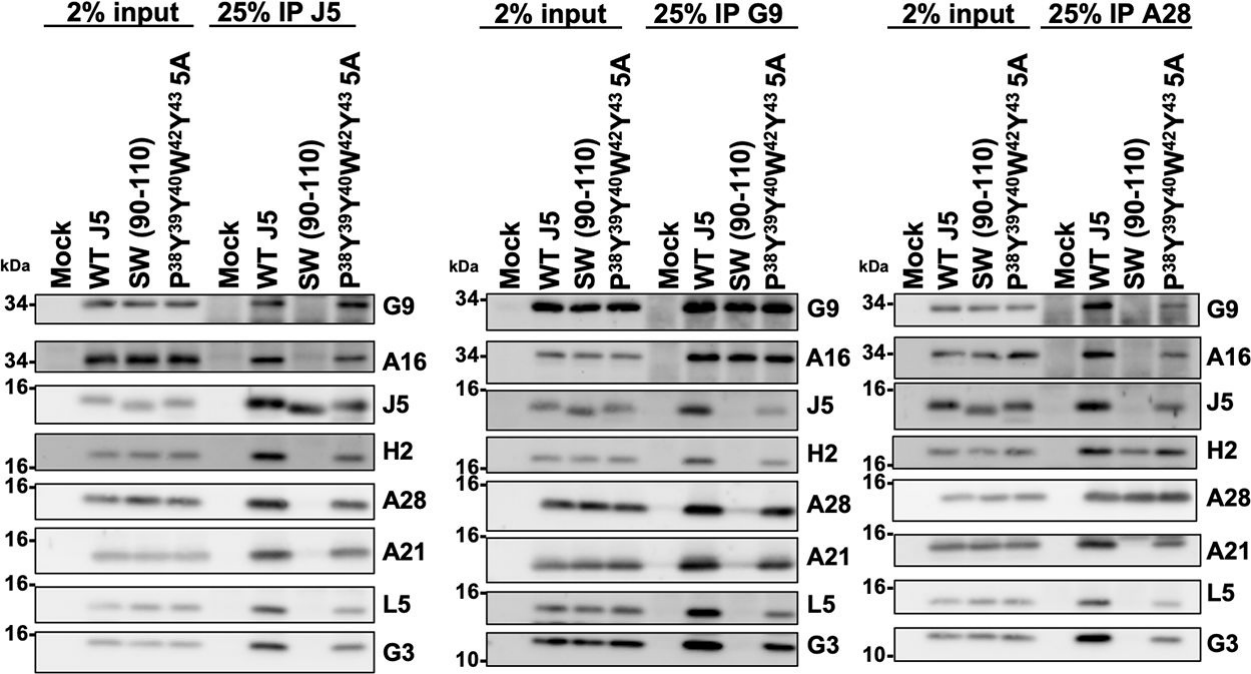
Distinct effects of J5 mutants on VACV EFC assembly. Immunoblots analysis of VACV EFC components following co-immunoprecipitation with antisera recognizing J5, G9, or A28. The left, middle, and right panels show immunoprecipitation with anti-J5, anti-G9, and anti-A28 antisera, respectively. Each panel includes Mock, WT J5, SW (90–110), and P^38^Y^39^Y^40^W^42^Y^43^5A mutant samples, with corresponding 2% input and 25% immunoprecipitation fractions. The SW (90–110) mutant failed to assemble a complete EFC but retained the A16/G9 and A28/H2 subcomplexes, whereas the J5 P^38^Y^39^Y^40^W^42^Y^43^5A mutant retained overall EFC assembly.

To determine the biochemical basis of the fusion defects, we performed co-immunoprecipitation (co-IP) analyses to examine EFC assembly in the two most severely impaired mutants, P^38^Y^39^Y^40^W^42^Y^43^5A and SW (90–110). As a control, WT J5 efficiently co-precipitated all EFC components when immunoprecipitated with anti-J5 antibody, confirming that the assay reliably reflects EFC assembly (Fig. 5, left panel). Based on the vaccinia EFC cryo-EM structure, the P^38^YYCWY^43^ motif resides within the helix α4 of J5 and mediates interactions with A28 and G9 through π–π stacking interactions between J5^W42^ and A28^F89^ and between J5^Y40^ and G9^Y237^ (43). Alanine substitution of these aromatic residues was predicted to destabilize these interactions and disrupt complex formation. Unexpectedly, the J5 P^38^Y^39^Y^40^W^42^Y^43^5A mutant retained interactions with most EFC components (Fig. 5, left panel), and reciprocal co-IP experiments with anti-G9 (Fig. 5, middle panel) and anti-A28 (Fig. 5, right panel) antibodies further supported that the overall EFC complex remained largely assembled. Thus, the severe defects in infectivity and membrane fusion observed for the P^38^Y^39^Y^40^W^42^Y^43^5A mutant may reflect that the P^38^YYCWY^43^ motif contributes to the fusion activation of the EFC.

In contrast, the J5 SW (90–110) failed to co-precipitate A16, G9, A28, H2, A21, L5, and G3 (Fig. 5, left panel). Reciprocal co-IPs using anti-G9 and anti-A28 antibodies showed that the A16/G9 (Fig. 5, middle panel) and A28/H2 subcomplexes (Fig. 5, right panel) remained intact, despite the absence of a full complex assembly. These results indicate that residues 90–110 are specifically required for incorporation of J5 into the central A16/G9/J5 trimer but are not required for the maintenance of individual EFC subcomplexes. Together, these findings revealed two functionally distinct elements within J5: the P^38^YYCWY^43^ motif is not required for EFC assembly but is essential for activation of membrane fusion, whereas the flexible region spanning residues 90–110 is required for stable incorporation of J5 into the EFC.

## DISCUSSION

**Fig 6:**
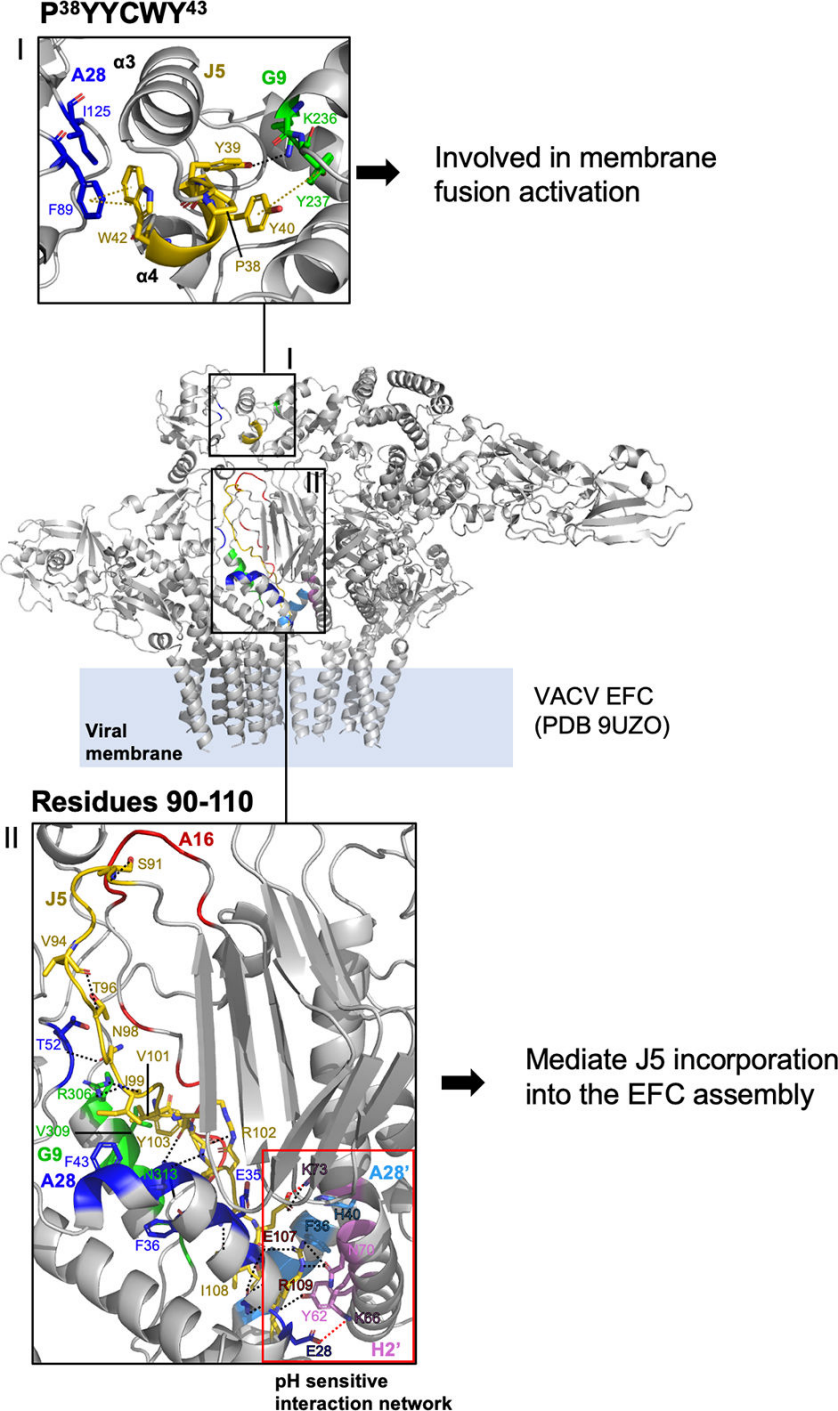
Summary of critical J5 residues and proposed model for EFC activation. Residue interaction network of the P^38^YYCWY^43^ motif (Inset 1) and residues 90–110 (Inset II) of J5 with interacting proteins located within 5 Å in the pre-fusion EFC cryo-EM structure (PDB 9UZO). The central panel shows the cryo-EM structure of VACV EFC (PDB 9UZO) sitting on the viral membrane layer. Interface residues are shown as sticks in two detailed close-up insets. Inset I shows the P^38^YYCWY^43^ motif involved in membrane fusion activation, highlighting its proximity to A28 (blue) and G9 (green). Inset II contains the dense, intricate interaction framework spanning residues 90–110 that tethers J5 to A16 (red), G9 (green), A28 (blue), and adjacent chains (A28′, H2′), outlining the proposed pH-sensitive interaction network (red box). Hydrogen bonds, salt bridges, and π–π interactions are indicated by dashed lines colored black, red, and yellow, respectively.

Vaccinia virus entry relies on the coordinated action of the EFC, a multiprotein machinery that is structurally and mechanistically distinct from classical viral fusion proteins (44–46). The cryo-EM structure of the pre-fusion vaccinia EFC was recently solved, revealing that J5 associates with G9 and A16 paralogs to form the central trimer (43). In this study, we demonstrate that the conserved P^38^YYCWY^43^ motif located within helix α4 and residues 90–110 of J5 are critical for viral infectivity. In addition, mutations in adjacent regions, including residues 22–40 on helix α3 and residues 70–88 corresponding to the Delta motif, also moderately impair EFC function. Mapping these functional sites onto the full-length J5 in the EFC complex (PDB 9UZO) provides a structural framework linking specific regions of J5 to the membrane fusion activity of the vaccinia EFC. In the pre-fusion EFC structure, the P^38^YYCWY^43^ motif of J5 interacts with A28 and G9 through π–π stacking and hydrogen bonding (Fig. 6, Inset I). Surprisingly, in the fusion-defective P^38^YYCWY^43^5A mutant virus, the EFC remained largely intact, suggesting that the P^38^YYCWY^43^ motif contributes to membrane fusion activation rather than EFC assembly. In a recent study, the AlphaFold-predicted model of the EFC suggested substantial rearrangements relative to the cryo-EM pre-fusion structure, particularly in the orientation of the A16/G9 N-terminus and the position of the J5 ectodomain (43). In this predicted model, the J5 ectodomain appears repositioned from the space between A28 and G9 toward the side of A16, which may allow the A16/G9 subcomplex to adopt a more upright conformation. Although the functional relevance of this predicted rearrangement remains unclear, it raises the possibility that the conserved P^38^YYCWY^43^ motif may participate in conformational transitions required for membrane fusion activation. Whether alanine substitution of this motif interferes with such rearrangements remains to be determined.

The second important functional region of J5, residues 90–110, forms extensive contacts with multiple EFC components, including the disordered loop of A16 and the pre-transmembrane helices of G9, A28, A28′, and H2′ (Fig. 6, Inset II). These interactions are primarily mediated by hydrogen bonds involving both backbone and side-chain atoms within residues 90–110 of J5 (black dashed lines in Inset II). The extensive contacts with multiple EFC proteins likely explain why the SW (90–110) mutant disrupted EFC assembly, as observed in the co-immunoprecipitation experiments. Notably, we identified a potential pH-sensitive interaction network centered on residues E107 and R109 of J5 (Fig. 6, red box in Inset II). At neutral pH, this region is predicted to be stabilized by two salt bridges, between J5^E107^ and H2′^K73^, and between A28^E28^ and H2′^K66^, thereby reinforcing local contacts among J5, A28, and H2′. In addition, A28′^H40^ lies within 3 Å of H2′^K73^, placing it in close proximity to both H2′^K73^ and J5^R109^. Upon acidification, protonation of histidine residues is predicted to render A28′^H40^ positively charged, potentially introducing electrostatic repulsion with H2′^K73^ and J5^R109^. At the same time, protonation of glutamate residues may disrupt salt bridges, weakening local interactions at this interface. Interestingly, a previously reported A28^H40AE44A^ mutant resulted in a loss of viral infectivity and impaired the ability of the EFC to trigger membrane fusion at low pH, which is consistent with this hypothesis (37). It is possible that these electrostatic changes could destabilize the local interface and potentially facilitate conformational rearrangements of the EFC during membrane fusion; however, whether residues 90–110 of J5 function as a pH-sensitive regulatory element remains to be experimentally tested in the future.

In summary, these findings identified two mechanistically distinct functional elements within J5 that regulate the assembly and activation of the vaccinia EFC. The P^38^YYCWY^43^ motif is dispensable for EFC assembly but required for membrane fusion activation, whereas the flexible region spanning residues 90–110 mediates interactions necessary for stable incorporation of J5 into the EFC.

## SUPPLEMENTAL MATERIAL

Supplemental figures jvi.00379-26-s0001.pdf Fig. S1 to S4.

Supplemental tables jvi.00379-26-s0002.pdf Tables S1 and S2.

ASM does not own the copyrights to Supplemental Material that may be linked to, or accessed through, an article. The authors have granted ASM a non-exclusive, world-wide license to publish the Supplemental Material files. Please contact the corresponding author directly for reuse.
