# Supplementary material for: Structure and functional analyses of vaccinia virus J5 protein reveal distinct determinants for entry-fusion complex assembly and activation
Source: J Virol. 2026 Jul 6;100(8):e00379-26. doi: 10.1128/jvi.00379-26 (PMC13483313; doi:10.1128/jvi.00379-26)
Supplement: Supplementary file 1 [file jvi.00379-26-s0001.pdf]

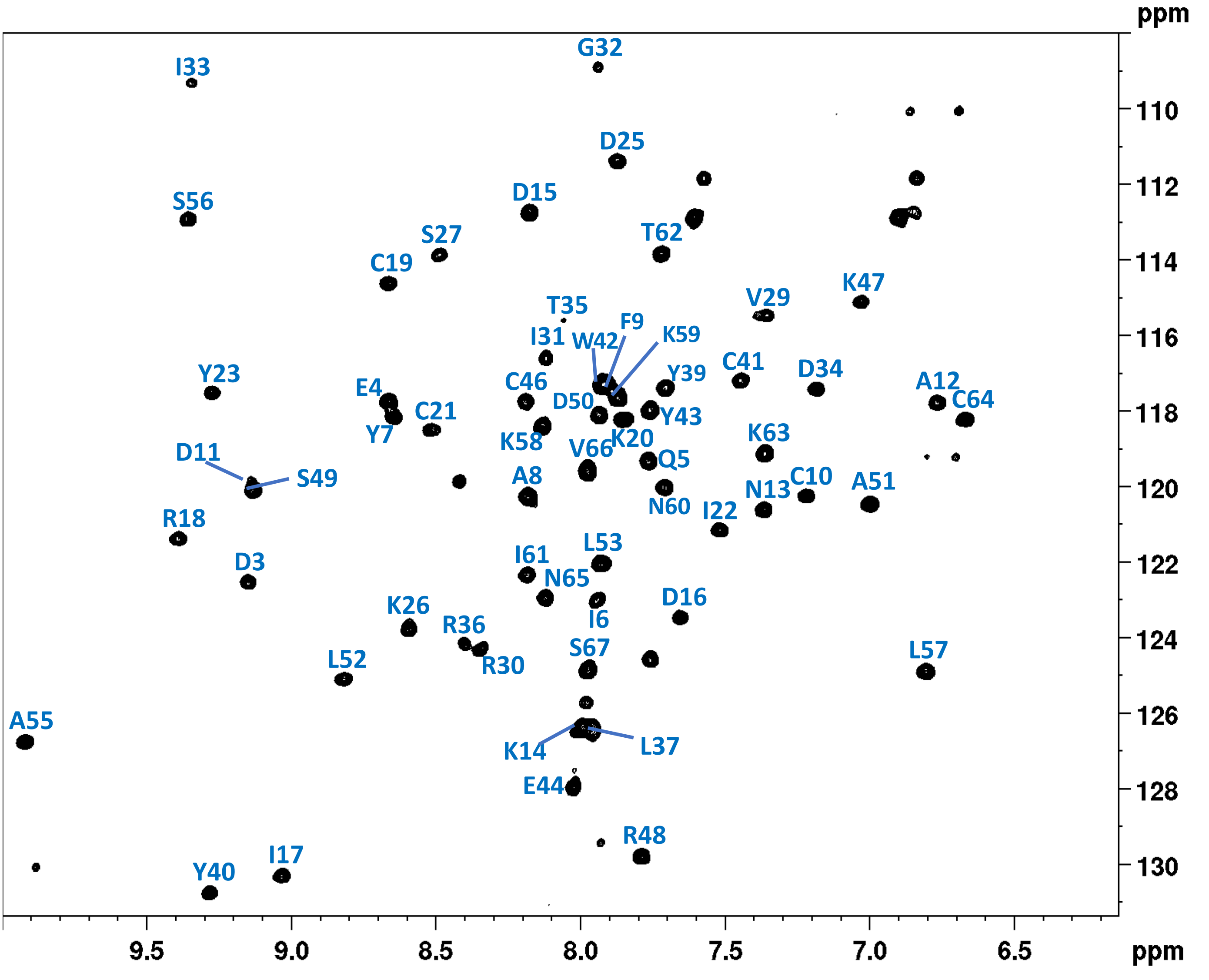

Fig. S1. 2D HSQC spectrum of vaccinia virus fusion component tJ5. 2D ^{1}H-^{15}N HSQC spectrum of N-isotope-labeled tJ5 (0.2 mM) at pH 6.5. The spectrum was collected at 25 °C in an NMR buffer containing 10 mM phosphate and 137 mM NaCl and 2.7 mM KCl. The assigned residues are indicated using single-letter codes.

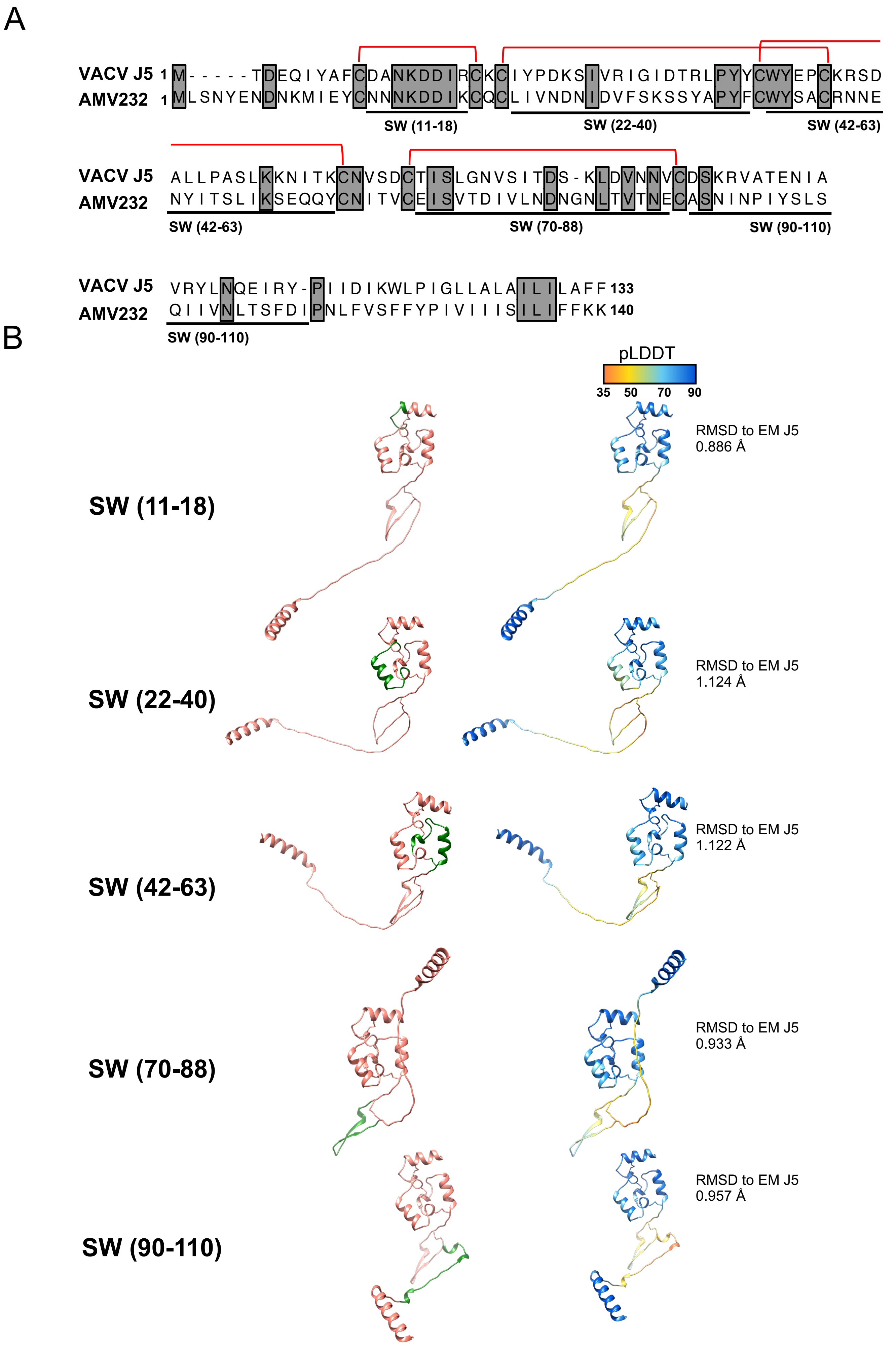
A
VACV J5 1 M-----TDEQIYAFCDANKDDIRCKCIYPDKSIVRIGIDTRLPYYCWYEPCKRSD
AMV232 1 MLSNYENDNKMIEYCNNNKDDIKCQCLIVNDNIDVFSKSSYAPYFCWYSACRNNE
SW (11-18)
SW (22-40)
SW (42-63)
VACV J5 ALLPASLKKNITKCNVSDCTISLGNVSITDS-KLDVNNVCDSKRVATENIA
AMV232 NYITSLIKSEQQYCNITVCEISVTDIVLNDNGNLTVTNECASNINPIYSLS
SW (42-63)
SW (70-88)
SW (90-110)
VACV J5 VRYLNQEIRY-PIIDIKWLPIGLLALAILILAFF 133
AMV232 QIIVNLTSFDIPNLFVSFFYPIVIIISILIFFKK 140
SW (90-110)
B
pLDDT
35 50 70 90
SW (11-18)
RMSD to EM J5 0.886 Å
SW (22-40)
RMSD to EM J5 1.124 Å
SW (42-63)
RMSD to EM J5 1.122 Å
SW (70-88)
RMSD to EM J5 0.933 Å
SW (90-110)
RMSD to EM J5 0.957 Å

Fig. S2. Design of J5 swap constructs and their predicted 3D models from AlphaFold2. (A) Sequence alignment of VACV J5 and AMV232, the J5 orthologue in *Amsacta moorei* entomopoxvirus. Identical residues are shaded in gray. Predicated disulfide bonds are indicated by red connecting lines. Swap (SW) regions between the two proteins are underlined. (B) Cartoon representation of the five swap mutant models. The left panel: AMV232 residues replaced in VACV J5 are highlighted in green, whereas WT J5 sequences are shown in salmon red. The right panel: Predicted local distance difference test (pLDDT) scores are mapped on the chimeric AlphaFold models. RMSD values relative to the cryo-EM J5 structure are indicated.

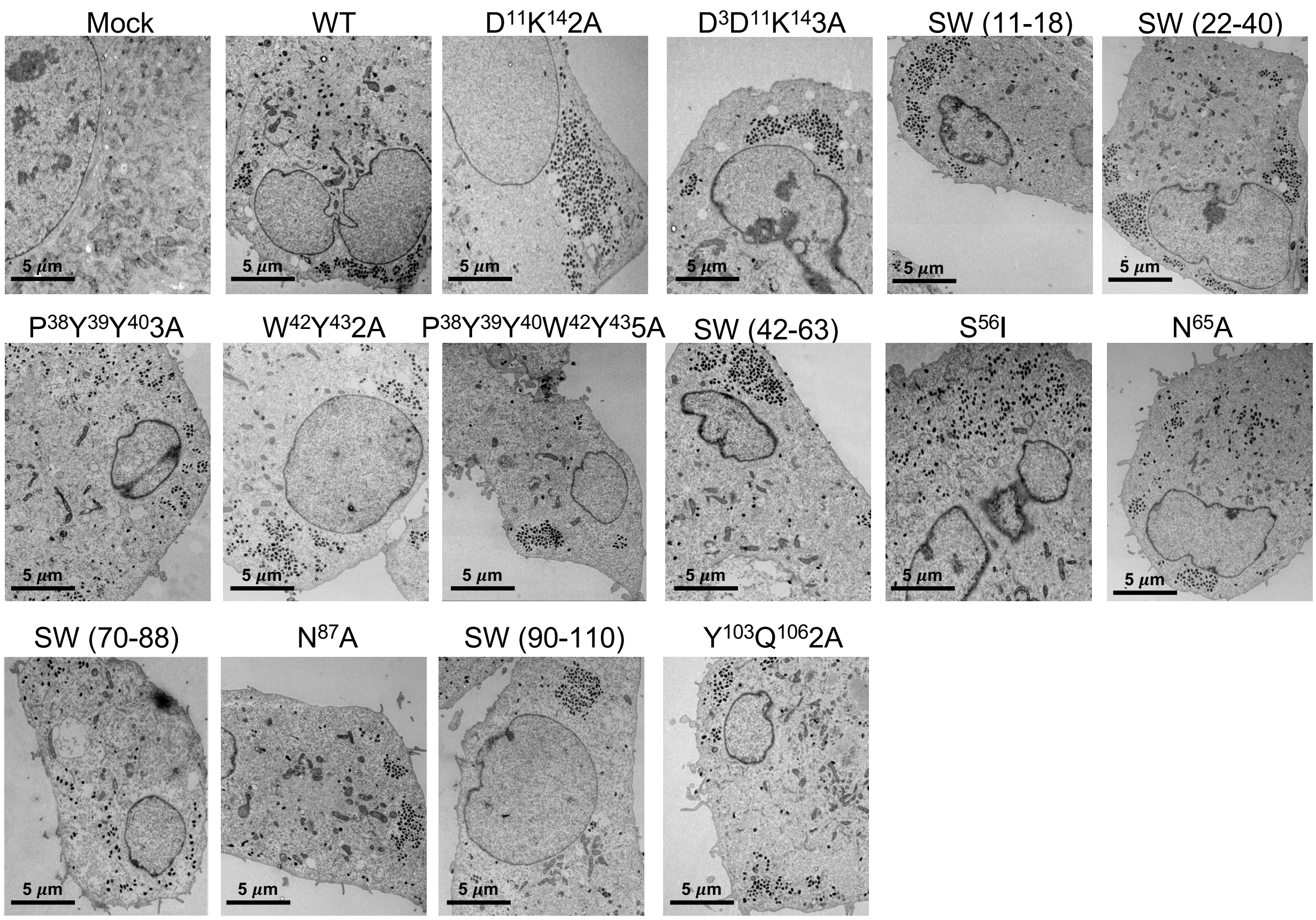

Fig. S3. Normal morphogenesis and MV production of WT and mutant J5 viruses. BSC40 cells were mock-infected, infected with WT or J5 mutant viruses for 24 h and subsequently processed for transmission electron microscopy as described in *Materials and Methods*. Scale bar, 5 μm.

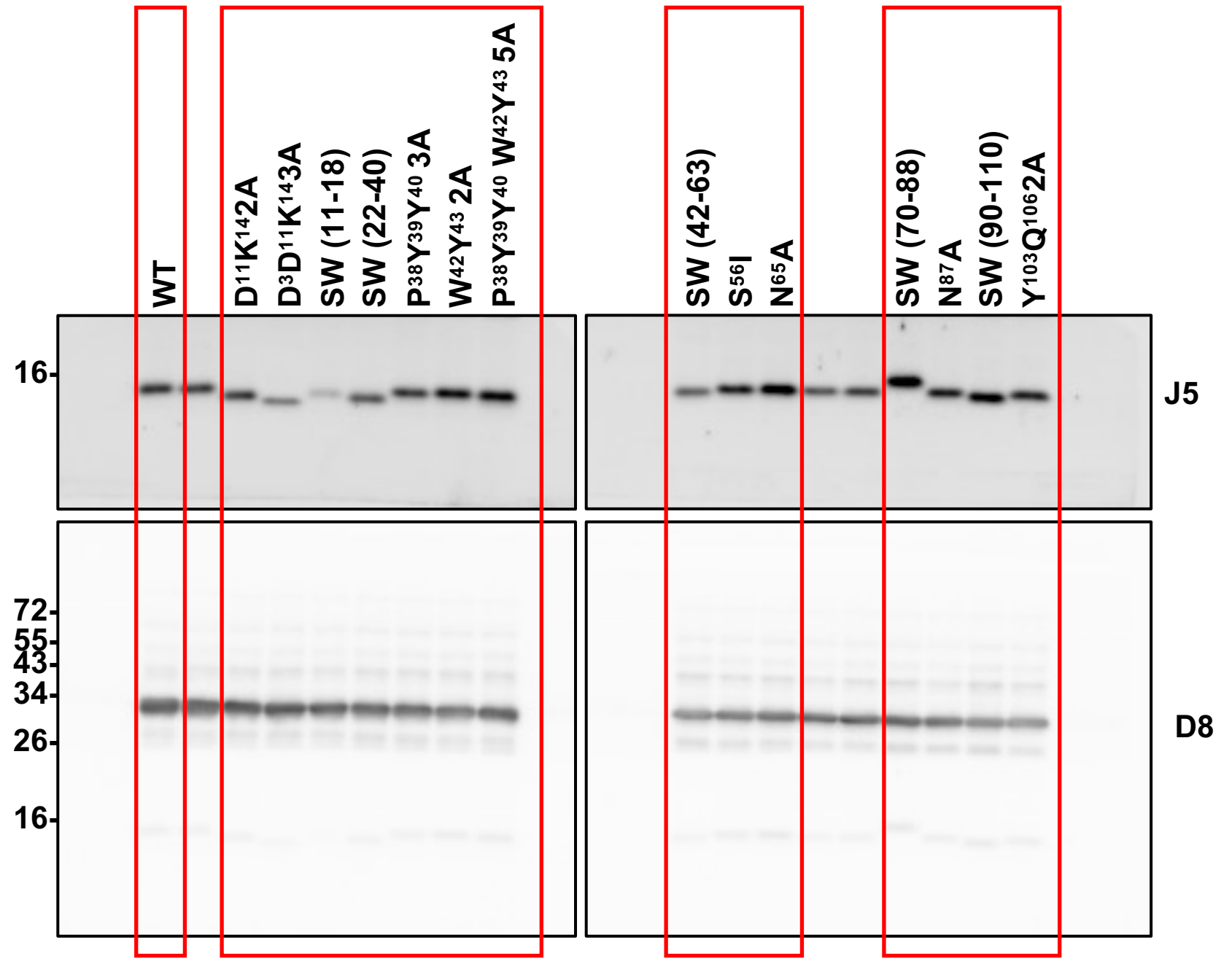

Fig. S4. Uncropped immunoblot images referred to Figure 3C. The red boxes indicate the lanes shown in Figure 3C.
