# Supplementary material for: Structure and functional analyses of vaccinia virus J5 protein reveal distinct determinants for entry-fusion complex assembly and activation
Source: J Virol. 2026 Jul 6;100(8):e00379-26. doi: 10.1128/jvi.00379-26 (PMC13483313; doi:10.1128/jvi.00379-26)
Supplement: Supplementary file 2 [file jvi.00379-26-s0002.pdf]

Table S1. List of construction strategies and sequence specifications of the J5 mutant plasmids.

No.	Mutant name	Mutant type	Plasmid Construction	Protein sequence [a]
1	SW (11-18)	Chimeric	Synthetic	MTDEQIYAFC**NNNKDDIK**CKCIYPDKSIVRIGID<br>TRLPYYCWYEPCKRSDALLPASLKKNITKCNVS<br>DCTISLGNVSITDSKLDVNNVCDSKRVATENIAV<br>RYLNQEIRYPIIDIKWLPIGLLALAILILAFF
2	SW (22-40)	Chimeric	Synthetic	MTDEQIYAFCDANKDDIRCKC**LIVNDNIDVFS**<br>**KSSYAPYF**CWYEPCKRSDALLPASLKKNITKCN<br>VSDCTISLGNVSITDSKLDVNNVCDSKRVATENI<br>AVRYLNQEIRYPIIDIKWLPIGLLALAILILAFF
3	SW (42-63)	Chimeric	Synthetic	MTDEQIYAFCDANKDDIRCKCIYPDKSIVRIGID<br>TRLPYYC**WYSACRNNENYITSLIKSEQQY**CN<br>VSDCTISLGNVSITDSKLDVNNVCDSKRVATENI<br>AVRYLNQEIRYPIIDIKWLPIGLLALAILILAFF
4	SW (70-88)	Chimeric	Synthetic	MTDEQIYAFCDANKDDIRCKCIYPDKSIVRIGID<br>TRLPYYCWYEPCKRSDALLPASLKKNITKCNVS<br>DC**EISVTDIVLNDNGNLTVTNE**CDSKRVATENI<br>AVRYLNQEIRYPIIDIKWLPIGLLALAILILAFF
5	SW (90-110)	Chimeric	Synthetic	MTDEQIYAFCDANKDDIRCKCIYPDKSIVRIGID<br>TRLPYYCWYEPCKRSDALLPASLKKNITKCNVS<br>DCTISLGNVSITDSKLDVNNVC**ASNINPIYSLSQ**<br>**IIVNLTSFDI**PIIDIKWLPIGLLALAILILAFF

6	$P^{38}Y^{39}Y^{40}$ 3A	Conserved	Synthetic	MTDEQIYAFCDANKDDIRCKCIYPDKSIVRIGID TRL**AAA**CWYEPCKRSDALLPASLKKNITKCNV SDCTISLGNVSITDSKLDVNNVCDSKRVATENIA VRYLNQEIRYPIIDIKWLPIGLLALAILILAFF
7	$W^{42}Y^{43}$2A	Conserved	Synthetic	MTDEQIYAFCDANKDDIRCKCIYPDKSIVRIGID TRLPYYC**AA**EPCKRSDALLPASLKKNITKCNVS DCTISLGNVSITDSKLDVNNVCDSKRVATENIAV RYLNQEIRYPIIDIKWLPIGLLALAILILAFF
8	$P^{38}Y^{39}Y^{40}$ $W^{42}Y^{43}$5A	Conserved	Synthetic	MTDEQIYAFCDANKDDIRCKCIYPDKSIVRIGID TRL**AAA**C**AA**EPCKRSDALLPASLKKNITKCNVS DCTISLGNVSITDSKLDVNNVCDSKRVATENIAV RYLNQEIRYPIIDIKWLPIGLLALAILILAFF
9	N^{65}A	Conserved	Synthetic	MTDEQIYAFCDANKDDIRCKCIYPDKSIVRIGID TRLPYYCWYEPCKRSDALLPASLKKNITKC**A**VS DCTISLGNVSITDSKLDVNNVCDSKRVATENIAV RYLNQEIRYPIIDIKWLPIGLLALAILILAFF
10	N^{87}A	Conserved	Synthetic	MTDEQIYAFCDANKDDIRCKCIYPDKSIVRIGID TRLPYYCWYEPCKRSDALLPASLKKNITKCNVS DCTISLGNVSITDSKLDVN**A**VCDSKRVATENIAV RYLNQEIRYPIIDIKWLPIGLLALAILILAFF
11	$D^{11}K^{14}$2A	Surface charged	Site-direct Mutagenesis [b]	MTDEQIYAFC**A**AN**A**DDIRCKCIYPDKSIVRIGID TRLPYYCWYEPCKRSDALLPASLKKNITKCNVS

				DCTISLGNVSITDSKLDVNNVCDSKRVATENIAV<br>RYLNQEIRYPIIDIKWLPIGLLALAILILAFF
12	$D^{3}D^{11}K^{14}$3A	Surface charged	Site-direct Mutagenesis [b]	MT**A**EQIYAFC**A**AN**A**DDIRCKCIYPDKSIVRIGID<br>TRLPYYCWYEPCKRSDALLPASLKKNITKCNVS<br>DCTISLGNVSITDSKLDVNNVCDSKRVATENIAV<br>RYLNQEIRYPIIDIKWLPIGLLALAILILAFF
13	S^{56}I	Conserved	Site-direct Mutagenesis[b]	MTDEQIYAFCDANKDDIRCKCIYPDKSIVRIGID<br>TRLPYYCWYEPCKRSDALLPA**I**LKKNITKCNVS<br>DCTISLGNVSITDSKLDVNNVCDSKRVATENIAV<br>RYLNQEIRYPIIDIKWLPIGLLALAILILAFF
14	$Y^{103}Q^{106}$2A	Conserved	Site-direct Mutagenesis [b]	MTDEQIYAFCDANKDDIRCKCIYPDKSIVRIGID<br>TRLPYYCWYEPCKRSDALLPASLKKNITKCNVS<br>DCTISLGNVSITDSKLDVNNVCDSKRVATENIAV<br>R**A**LN**A**EIRYPIIDIKWLPIGLLALAILILAFF

[a] Protein residues in bold and underlined indicate the mutated segments introduced through gene synthesis or site-directed mutagenesis (as described in the text), whereas unmarked residues correspond to the WT J5 protein sequences.

[b] Primers used for the construction of these point mutants are listed in Table S2.

Table S2. List of primers used to construct J5 mutants.

Mutation		Primer sequences 5'-3'
$D^{11}K^{14}2A$	F	CAA ATT TAT GCA TTC TGT GCT GCT AAC GCA GAC GAT ATA CGA TGT AA
	R	TTA CAT CGT ATA TCG TCT GCG TTA GCA GCA CAG AAT GCA TAA ATT TG
$D^{3}D^{11}K^{14}3A$ (Derived from $D^{11}K^{14}2A$)	F	TAA ATT TTT ATC ATG ACG GCT GAA CAA ATT TAT GCA TTC
	R	GAA TGC ATA AAT TTG TTC AGC CGT CAT GAT AAA AAT TTA
$S^{56}I$	F	GAT GCG TTG TTA CCA GCC ATT TTA AAA AAA AAT ATA AC
	R	GTT ATA TTT TTT TTT AAA ATG GCT GGT AAC AAC GCA TC
$Y^{103}Q^{106}2A$	F	GAG AAT ATA GCT GTC CGC GCT CTG AAT GCA GAA ATT AGA TAC CCT ATT
	R	AAT AGG GTA TCT AAT TTC TGC ATT CAG AGC GCG GAC AGC TAT ATT CTC
